# TPH-2 Gene Polymorphism in Major Depressive Disorder Patients With Early-Wakening Symptom

**DOI:** 10.3389/fnins.2018.00827

**Published:** 2018-11-15

**Authors:** Shiwan Tao, Mohammad Ridwan Chattun, Rui Yan, Jiting Geng, Rongxin Zhu, Junneng Shao, Qing Lu, Zhijian Yao

**Affiliations:** ^1^Department of Psychiatry, The Affiliated Brain Hospital of Nanjing Medical University, Nanjing, China; ^2^School of Biological Sciences and Medical Engineering, Southeast University, Nanjing, China; ^3^Key Laboratory of Child Development and Learning Science, Southeast University, Nanjing, China; ^4^Medical School of Nanjing University, Nanjing Brain Hospital, Nanjing, China

**Keywords:** circadian disruption, early wakening, major depressive disorder, resting-state functional magnetic resonance imaging, tryptophan hydroxylase-2

## Abstract

**Background:** Sleep disturbances, such as early wakening, are frequently observed in patients with major depressive disorder (MDD). The suprachiasmatic nuclei (SCN), which controls circadian rhythm, is innervated by the raphe nucleus, a region where Tryptophan hydroxylase-2 (TPH-2) gene is primarily expressed. Although TPH-2 is often implicated in the pathophysiology of depression, few studies have applied a genetic and imaging technique to investigate the mechanism of early wakening symptom in MDD. We hypothesized that TPH-2 variants could influence the function of SCN in MDD patients with early wakening symptom.

**Methods:** One hundred and eighty five MDD patients (62 patients without early wakening and 123 patients with early wakening) and 64 healthy controls participated in this study. Blood samples were collected and genotyping of rs4290270, rs4570625, rs11178998, rs7305115, rs41317118, and rs17110747 were performed by next-generation sequencing (NGS) technology. Logistic regression model was employed for genetic data analysis using the PLINK software. Based on the allele type, rs4290270, which was significant in the early wakening MDD group, participants were categorized into two groups (A allele and T carrier). All patients underwent whole brain resting-state functional magnetic resonance imaging (rs-fMRI) scanning and a voxel-wise functional connectivity comparison was performed between the groups.

**Results:** rs4290270 was significantly linked to MDD patients who exhibited early wakening symptom. The functional connectivities of the right SCN with the right fusiform gyrus and right middle frontal gyrus were increased in the T carrier group compared to the A allele group. In addition, the functional connectivities of the left SCN with the right lingual gyrus and left calcarine sulcus were decreased in the T carrier group compared to the A allele group.

**Conclusion:** These findings suggested that the TPH-2 gene variant, rs4290270, affected the circadian regulating function of SCN. The altered functional connectivities, observed between the SCN and right fusiform gyrus, right middle frontal gyrus, the right lingual gyrus and left calcarine sulcus, could highlight the neural mechanism by which SCN induces sleep-related circadian disruption in T carrier MDD patients. Hence, rs4290270 could potentially serve as a reliable biomarker to identify MDD patients with early wakening symptom.

## Introduction

Major depressive disorder (MDD) is a neuropsychiatric disorder that affects over 300 million individuals globally (World Health Organization [WHO], 2017). MDD has a heritability of 31–42%, (Major Depressive Disorder Working Group of the Psychiatric Genomics Consortium, 2012) and is considered to be a circadian-related illness as it is accompanied by altered biological processes such as sleep, temperature, mood, and hormone secretion (Bunney et al., 2015).

The most commonly and robustly observed circadian rhythm disruption in MDD patients is sleep disturbances (Vadnie and McClung, 2017). Sleep-related circadian disruption include delayed sleep phase, advanced sleep phase, non-24-h sleep/wake cycles, disorganized sleep/wake patterns, shift work, and jet lag (Dagan, 2002). Altered sleep phase such as early sleep-onset and early-morning wakening has also commonly been observed in depression (Hickie and Rogers, 2011). MDD patients regularly presented with a phase shift, disrupted circadian synchronization, and lower amplitude of circadian genes (Edgar and McClung, 2013). The desynchronization of internal circadian rhythms with the external environment could bring about alterations in mood (Wirz-Justice, 2003). Additionally, the severity of depression is correlated with the extent of circadian rhythms desynchronization (Bunney et al., 2015). An internal misalignment of the master circadian pacemaker could lead to inappropriately timed sleep and waking up too early (Baron and Reid, 2014).

The timing of our internal biological clock has always been a major concern in the pathogenesis of MDD. MDD patients frequently complain that their sleep cycle has been prematurely terminated early in the morning. It was previously reported that almost 90% of MDD patients had difficulty in initiating and maintaining sleep (Soehner et al., 2014). Early wakening was the sleep symptom that most consistently related to depressed mood. Among 1253 first-episode and recurrent MDD patients, 30.3% had early morning wakening (Saletu-Zyhlarz et al., 2002). Early wakening patients suffer from both partial insomnia and sleep deprivation since they have difficulty in maintaining sleep, and as a consequence have insufficient sleep. Since the total sleep duration of sleep need is inadequate, this results in daytime fatigue and reduced productivity (Buysse, 2013). Some types of depression namely “typical” or “melancholic” have discernible changes in diurnal mood variation, which give rise to an increased risk of early wakening (Hickie et al., 2013). Even though waking up early is the most typical symptom of depressed patients, not much attention has been paid to this chronobiologic abnormality.

Circadian rhythm is adjusted by light-dark exposure over a 24-h period in the anterior portion of the suprachiasmatic nuclei (SCN), which is the “master clock” or internal biological time-keeping system (Vadnie and McClung, 2017). The SCN orchestrates the synchronization of circadian rhythm, and maintains the timing of sleep and wakefulness throughout the day (Reichert et al., 2017). The SCN not only acts as integrator to the classical light-affected retinohypothalamic tract (RHT) but also receives serotonergic innervations from the median raphe nucleus (Morin, 2013). Ocular light, which passes through the retinal ganglion cells, is a predominant cue for the mediation of circadian rhythm that entrains the activity of SCN (Skene et al., 1999). An abnormality in the SCN function disrupted the circadian rhythm and caused fluctuations in the regular sleep architecture (Hickie et al., 2013). The SCN comprises of numerous genes and encoded protein products which are responsible for normal circadian clock function (Vitaterna et al., 2001).

Tryptophan hydroxylase-2 (TPH-2) is a protein coding gene that belongs to the biopterin-dependent aromatic amino acid hydroxylase family and is the rate-limiting isozyme in the biosynthesis of neuronal serotonin (5-HT) (Zhang et al., 2004). TPH-2 gene was found to be primarily expressed in the serotonergic cells of the raphe nucleus (Bach-Mizrachi et al., 2006). TPH-2 has been widely debated as a major candidate gene in numerous psychiatric disorders including MDD, anxiety, schizophrenia, and bipolar disorder (Zill et al., 2004). Numerous candidate TPH-2 variants, namely rs4290270 (Shen et al., 2011; Wang et al., 2015; Karanovic et al., 2017; Mei et al., 2018), rs4570625 (Gao et al., 2012; Mandelli et al., 2012; Lehto et al., 2015; Han et al., 2017; Piel et al., 2018; Wigner et al., 2018), rs41317118 (Xu et al., 2013), rs11178998 (Xu et al., 2016; Ottenhof et al., 2018), rs7305115 (Ke et al., 2006; Van der Auwera et al., 2014; Wang et al., 2015; Ottenhof et al., 2018), rs17110747 (Tsai et al., 2009; Ottenhof et al., 2018), rs10748185 (Serretti et al., 2011; Ottenhof et al., 2018), rs18438099 (Anttila et al., 2009; Ottenhof et al., 2018), rs11316791, rs1386493, rs1386494 (Haghighi et al., 2008), have been prominently associated with MDD. The exonal variants, rs7305115 and rs4290270, were prominently associated with MDD and disturbed sleep (Utge, 2012; Mei et al., 2018).

Tryptophan hydroxylase-2 gene polymorphisms have demonstrated strong epidemiological associations with MDD (Gao et al., 2012). The genetic polymorphisms involved in TPH-2 gene expression could alter the physiological processes of 5-HT. A decrease in 5-HT was associated with an altered pattern of circadian regulation and sleep-wake homeostasis (Whitney et al., 2016). It is of crucial importance to determine the functional consequences of single nucleotide polymorphisms (SNPs) as it could affect TPH-2 gene expression and function. Previous studies have observed that TPH-2 mRNA and TPH-2 protein levels are correlated with rhythmic variation in raphe nuclei, and imposed an opposite phase in the SCN (Malek et al., 2004). It was also reported that 5-HT is involved in the mechanism of circadian rhythm regulation in the SCN (Morin, 2013). Therefore, it is plausible that an imbalance in 5-HT levels could occur due to TPH-2 variations. The changes in 5-HT neurotransmission could play a role in the etiology of depression and lead to sleep troubles.

Waking up early is believed to occur a result of complex interaction between serotonergic and other neurotransmitter systems. The intertwining serotonin-circadian interaction is of crucial importance for the understanding of the genetic basis of depression. 5-HT neurotransmission regulates diverse physiological and behavioral functions including mood, behavior, appetite, sleep, and memory (Martinowich and Lu, 2007). There is an overlapping relationship between 5-HT neurotransmission and circadian rhythm disturbances in the brain (Ciarleglio et al., 2011). In the past, researchers have reported that 5-HT reset, phase shift and modulate the circadian rhythms in the SCN (Lovenberg et al., 1993). TPH-2 gene polymorphisms could cause a deficient 5-HT neurotransmission due to reduced tryptophan uptake, TPH-2 hypofunction or decreased 5-HT neuronal firing (Jacobsen et al., 2011). However, researchers have not fully understood the underlying functional mechanism through which TPH-2 variants affect circadian rhythm synchronization in the SCN.

To date, genetic and genomic researches have had limited success at identifying reliable and distinct genes which could be attributed to circadian disruption in depression. Since MDD is regarded as a highly heterogeneous disease consisting of similar clinical phenotypes arising from multiple etiopathological mechanisms, a deeper investigation of circadian-related symptomatic homogeneity, such as early wakening and non-early wakening, could potentially identify reliable genetic markers of MDD (Major Depressive Disorder Working Group of the Psychiatric Genomics Consortium, 2012). The neuroimaging of the behavioral symptoms is a valuable technique to study the functional effects of specific genes associated with diseases. Resting-state functional magnetic resonance imaging (rs-fMRI) has the ability to measure the temporal correlation between brain areas. The use of rs-fMRI is suitable for investigating the functional connectivity of SCN because it can measure the regional differences in cerebral blood flow using blood-oxygen-level dependent (BOLD) contrast imaging. In the current study, we investigated the relationship of TPH-2 gene variants with early wakening symptom in MDD and explored the functional connectivity of SCN in the whole brain.

## Materials and Methods

### Participants

A total of 249 subjects were recruited from the Affiliated Brain Hospital of Nanjing Medical University between September 2014 and December 2017. One hundred and eighty five patients were diagnosed with MDD by at least two attending physicians using the Mini-International Neuropsychiatric Interview (MINI) (Hergueta et al., 1998), according to the criteria the Diagnostic and Statistical Manual of Mental Disorders, Fourth Edition, Text Revision (DSM-IV-TR; American Psychiatric Association, APA) (Association and Association, 2000). Patients were also assessed with the 24-item Hamilton Rating Scale for Depression (HAMD-24). MRI scanning was performed within 2 days of hospitalization. In order to reduce the probability of diagnostic errors, all patients were evaluated with the 32-item Hypomania/Mania Symptom Checklist (HCL-32) to exclude mania or hypomania episodes. For patients who presented with atypical symptoms or a HAMD-24 score of <17, a clinical interview, in accordance with the HCL-32 scale, was made by a chief physician. MDD patients were divided into two groups based on score of the six-item version of the Hamilton Depression Scale (HAMD-6) namely, 0 for not waking early in the morning (NWE, *n* = 62) and 1 or 2 for waking early in the morning (WE, *n* = 123). Sixty four healthy controls, matched in age, gender, and marriage were enrolled via word-of-mouth and local hospital advertisements.

### Ethics Statement

This study abided by the ethical guidelines of the World Medical Association Declaration of Helsinki (World Medical Association, 2013) and was approved by the Medical Research Ethics Review Board of the Affiliated Brain Hospital of Nanjing Medical University. All subjects have signed an informed consent agreement.

### Inclusion and Exclusion Criteria

All participants were right-handed, Chinese and aged >18 and <55 years old. Patients did not undergo any physical therapies such as repetitive transcranial magnetic stimulation (rTMS) and electroconvulsive therapy (ECT) within the past 6 months before the scan. None of the included patients had any contraindication to perform MRI scanning. Patients with a history of alcohol and substance abuse were excluded. Patients who presented with organic brain diseases, severe somatic diseases, neurological diseases and comorbid psychiatric illnesses were ruled out. Healthy controls were screened for any medical conditions and adopted similar exclusion criteria to that of patients. Healthy controls with first-degree relatives having any DSM-IV Axis-1 psychiatric disorders were also discarded.

### Single-Nucleotide Polymorphisms Selection Criteria

The candidate SNPs of TPH-2 gene met with a minimum of two criteria from the following: (1) The SNPs must have been previously associated with MDD or a depression episode; (2) The SNPs were located in the UTR3, UTR5, or CDS region within TPH-2 gene; and (3) A minor allele frequency of >5% in Han Chinese population. As a result, 6 SNPs were selected for analysis.

### Genotyping Process

In order to determine TPH-2 gene variants in all subjects, genotyping analysis was performed according to the following steps:

(1) Venous blood collection: 2 ml of peripheral venous blood samples were collected from every participant and were stored in EDTA-containing tubes at -80°C.
(2) Blood DNA extraction: Genomic DNA was extracted using QiAamp DNA Blood Midi Kit (Qiagen, Germany). DNA concentration was quantified by Qubit dsDNA HS Assay Kit (Invitrogen, United States).
(3) PCR primer design: 500 base pairs (bp) 5′-flanking sequences for each SNPs were extracted from the dbSNP database^1^. PCR primers were designed using Primer Premier 5 software (Premier Biosoft, United States) under default settings. The length of the primers ranged from 18 to 27 bp and the melting temperature (Tm) was 57–62°C.
(4) Multiplex PCR amplification: Multiplex PCR amplification was performed with five pairs of primers and 20 ng of genomic DNA template using Premix TaqTM kit (Ex TaqTM Version 2.0) (Takara, JAPAN). The PCR program was as follows: 95°C for 2 min; 15 cycles of 95°C for 30 s, 60°C for 2 min, 72°C for 2 min; and 72°C for 5 min. The PCR products were then purified using AMpure XP magnetic beads (Beckman-Coulter, United States). NEXTflex Rapid DNA-seq kit (Bioo Scientific, United States) was used to construct a sequencing library which was then subjected to Illumina HiSeq X-10 sequencing system (Illumina, United States) for DNA sequencing.
(5) Sequencing data analysis: Sequencing data were evaluated with SOAPnuke, and processed by cutadapt (v1.11) to remove primer sequences. Alignment to the human genome (GRCh37/hg19) was performed using BWA-MEM (v0.7.5). Thereafter, Picard (v1.85)^2^ was employed for data format conversion. GATK (v2.6-5) was then used for local indel realignment and base quality recalibration. Finally, GATK UnifiedGenotyper was utilized for SNP calling and identification, with a depth of <10 or a genotype quality of <20.

### fMRI Data Acquisition

The participants were instructed to lie in the supine position, eyes-closed, in a relaxed state and not fall asleep. All participants underwent a MRI scan with a 3T Siemens Verio scanner using a gradient-recalled echo-planar imaging (GRE-EPI) with parameters including echo time (TE) = 40 ms, repetition time (TR) = 3000 ms, flip angle = 15°, slice number = 32, slice thickness = 4 mm, slice gap = 4 mm, field of view (FOV) = 240 mm × 240 mm, matrix size = 64 × 64, in-plane voxel resolution = 3.75 mm × 3.75 mm, volume number = 133, and a total duration of resting-state scans for 6 min 45 s. High resolution T1-weighted 3D structural images were obtained with parameters including TR = 1900 ms, TE = 2.48 ms, flip angle = 9°, slice number = 176, slice thickness = 1 mm, in-plane voxel resolution = 1 mm × 1 mm, FOV = 250 mm × 250 mm, matrix size = 256 × 256. The data acquisition lasted for 4 min 18 s.

### Data Pre-processing and Analysis

Resting-state functional magnetic resonance imaging data were pre-processed and analyzed by MRIcroN^3^, MATLAB R2012a (Mathworks Inc., MA, United States) and Data Processing Assistant for Resting-State fMRI (DPARSF) toolbox^4^. MRIcroN converted the format of scanned images and DPARSF preprocessed the converted images. The first six volumes of each subject were removed. The remaining volumes underwent slice-time correction, head motion correction, co-registration, spatial normalization (resampled to 2 mm × 2 mm × 2 mm voxels). Data also underwent temporal band-pass filter (0.01–0.08 Hz), linear detrend and segmentation of white matter as well as cerebrospinal fluid. Head movements was restricted to <2 mm in any direction of x, y, and z and <2° in any angular dimension. A total of 158 subjects satisfied the quality control criteria and was used for further analysis.

Functional connectivity analysis was performed on all MDD patients using the DPARSF toolbox. The regions of interests (ROIs) for the seed region were two 2-mm radius spheres, centered at 3, 5, -8 (right SCN) and -2, 5, -8 (left SCN) (Vimal et al., 2009). The data from all voxels in each ROI was averaged to obtain the time-series of the seed region. Thereafter, Pearson’s correlation was performed between the averaged time series of all voxels in each ROI and the time series of all voxels from the whole brain. In order to improve normalization, correlation coefficients were transformed into *z*-values using Fisher’s transformation.

### Gene Data Analysis

The distribution for age, years of education, marriage, and gender of different groups (MDD, HC; NWE, HC; WE, HC) were compared using the independent-sample *t*-test and chi-square test in SPSS 19.0 software (SPSS Inc., Chicago, IL, United States). Significance threshold was set at *p*-value < 0.05. Except for years of education and family history, there were no significant difference between any groups. Variables including age, years of education, gender, marriage, and family history, which are regarded as risk factors of MDD, were added as covariates.

The PLINK v1.07 software was used to analyze the individual genotyping call rate, minor allele frequency (MAF), and Hardy-Weinberg equilibrium (HWE) for each SNP. SNPs with missing call rate >10%, *p* < 0.05 of HWE or MAF < 0.05 were excluded. In case-control analysis, the remaining SNPs were tested with logistic regression model in order to investigate the association with MDD (Bonferroni corrected and adjusted for age, gender, education, marriage, and family history). Patients were assigned into either the NWE or WE group in the same model based on their HAMD-6 score and the statistically significant SNP was used for functional connectivity analysis.

### Functional Connectivity Analysis

According to the significant result of the genetic data analysis (rs4290270), patients were divided into two groups (A allele and T allele carrier). There was no significant difference in age, years of education, and gender between the two groups.

The functional connectivity maps of the two groups were compared by using independent-sample *t*-test in the Resting-State fMRI Data Analysis Toolkit (REST) software^5^, with age, years of education and gender applied as covariates. The significant threshold was set at *p*-value <0.001, and a minimum cluster size of 48 mm^3^. The parameters were obtained by the cluster threshold size estimator plug-in in the REST software. AlphaSim correction was used for multiple comparisons with the following parameters: individual voxel *p*-value <0.001, minimum cluster size of 48 mm^3^, Full width at half maximum (FWHM) 4 mm, which corresponded to a corrected *p* < 0.05.

## Results

### Genetic Analyses

There were no significant differences among the groups (MDD and HC; NWE and HC; WE and HC) in terms of age, marriage, and gender. However, for each group, the years of education differed significantly. The *p*-values for gender and marriage were obtained by chi-square test whereas the ones for age and years of education were calculated by independent sample *t*-test. Subjects’ demographic characteristics are shown in Table 1.

**Table 1:** Demographic characteristics of subjects.

Variables		MDD (*n* = 185)	HC (*n* = 64)	*p*-value	NWE (*n* = 62)	HC (*n* = 64)	*p*-value	WE (*n* = 123)	HC (*n* = 64)	*p*-value
Gender (male/female)^a^		73/112	29/35	0.41	29/33	29/35	0.87	44/79	29/35	0.21
Family history (with/without)		44/141	0/64	–	13/49	0/64	–	31/92	0/64	–
Marriage (married/not married)^a^		109/76	37/27	0.88	31/31	37/27	0.38	78/45	37/27	0.46
Age (years)^b^		33.38 ± 10.32	32.97 ± 9.78	0.78	31.47 ± 10.05	32.97 ± 9.78	0.40	34.34 ± 10.36	32.97 ± 9.78	0.38
Education (years)^b^		13.48 ± 3.06	15.30 ± 2.86	0.00	13.65 ± 2.80	15.30 ± 2.86	0.00	13.39 ± 3.19	15.30 ± 2.86	0.00

*MDD, major depression disorder; NWE, non-waking early; WE, waking early; HC, healthy control.*^a^*The *p*-value was obtained by two-tailed chi-square test.*^b^*Data is presented as mean ± standard deviation. The *p*-value was obtained by two-tailed independent *t*-test*.

All selected SNPs distributions were in HWE (rs4290270: *p* = 0.1395; rs4570625: *p* = 0.7942; rs11178998: *p* = 0.6719; rs7305115: *p* = 0.1291; rs41317118: *p* = 1; rs17110747: *p* = 1), and no SNPs had a call rate of >10% and MAF < 0.05. The allele frequency for each group was >0.05. The correlations between MDD and HC, NWE and HC, WE and HC groups (Bonferroni corrected, *p* threshold = 0.0083, adjusted by gender, age, marriage, years of education, and family history) were then assessed. There was no significant result between the MDD and HC group (rs4290270: *p* = 0.0167; rs4570625: *p* = 0.6236; rs11178998: *p* = 0.7626; rs7305115: *p* = 0.2814; rs41317118: *p* = 0.9857; rs17110747: *p* = 0.7617). However, rs4290270 showed a significant association in the WE group [*p* = 0.0047; OR = 2.027 (1.242–3.308); associating allele: T] but not in the NWE group [*p* = 0.175; OR = 1.489 (0.8376–2.648)]. The detailed allele frequency, covariate-adjusted odds ratio and 95% confidence interval are depicted in Table 2.

**Table 2:** Genotyping of TPH-2 variants.

Variant		MDD	HC	OR (95% CI)	*p*-value	NWE	HC	OR (95% CI)	*p*-value	WE	HC	OR (95% CI)	*p*-value	HWE
rs4290270	T	185 (0.4162)	64 (0.5469)	1.716 (1.103–2.669)	0.0167	62 (0.4597)	64 (0.5469)	1.489 (0.838–2.648)	0.1750	123 (0.3943)	64 (0.5469)	2.027 (1.242–3.308)	0.0047	0.1395
	A	185 (0.5838)	64 (0.4531)	1.00 (ref)		62 (0.5403)	64 (0.4531)	1.00 (ref)		123 (0.6057)	64 (0.4531)	1.00 (ref)		
rs4570625	G	185 (0.4915)	64 (0.5254)	1.115 (0.722–1.730)	0.6236	62 (0.5263)	64 (0.5254)	1.174 (0.688–2.005)	0.5554	123 (0.5000)	64 (0.5254)	1.075 (0.668–1.732)	0.7659	0.7942
	T	185 (0.5085)	64 (0.4746)	1.00 (ref)		62 (0.4737)	64 (0.4746)	1.00 (ref)		123 (0.5000)	64 (0.4746)	1.00 (ref)		
rs11178998	G	185 (0.1595)	64 (0.1797)	1.087 (0.634–1.863)	0.7626	62 (0.1855)	64 (0.1797)	0.971 (0.485–1.943)	0.9335	123 (0.1463)	64 (0.1797)	1.181 (0.650–2.146)	0.5855	0.6719
	A	185 (0.8405)	64 (0.8203)	1.00 (ref)		62 (0.8145)	64 (0.8203)	1.00 (ref)		123 (0.8537)	64 (0.8203)	1.00 (ref)		
rs7305115	G	185 (0.4703)	64 (0.5391)	1.269 (0.823–1.956)	0.2814	62 (0.5161)	64 (0.5391)	1.213 (0.695–2.117)	0.4977	123 (0.4634)	64 (0.5391)	1.350 (0.838–2.178)	0.2178	0.1291
	A	185 (0.5297)	64 (0.4609)	1.00 (ref)		62 (0.4839)	64 (0.4609)	1.00 (ref)		123 (0.5366)	64 (0.4609)	1.00 (ref)		
rs41317118	A	185 (0.0703)	64 (0.0625)	0.992 (0.391–2.514)	0.9857	62 (0.0645)	64 (0.0625)	0.787 (0.240–2.583)	0.6925	123 (0.0732)	64 (0.0625)	0.981 (0.364–2.643)	0.9691	1
	G	185 (0.9297)	64 (0.9375)	1.00 (ref)		62 (0.9355)	64 (0.9375)	1.00 (ref)		123 (0.9268)	64 (0.9375)	1.00 (ref)		
rs17110747	A	185 (0.2450)	64 (0.2188)	0.920 (0.536–1.580)	0.7617	62 (0.2177)	64 (0.2188)	0.976 (0.500–1.903)	0.9420	123 (0.2520)	64 (0.2188)	0.854 (0.476–1.533)	0.5971	1
	G	185 (0.7550)	64 (0.7812)	1.00 (ref)		62 (0.7823)	64 (0.7812)	1.00 (ref)		123 (0.7480)	64 (0.7812)	1.00 (ref)		

*MDD, major depression disorder; NEW, non-waking early; WE, waking early; HC, healthy control; HWE: Hardy–Weinberg–Equilibrium*.

### Functional Connectivity Analyses

All depressed patients were divided into two groups including those with allele A (*n* = 58, mean age ± SD = 34.40 ± 11.23 years old, mean education ± SD = 13.09 ± 2.98 years, mean HAMD score = 27.90 ± 7.01) and those with allele T (*n* = 100, mean age ± SD = 32.60 ± 9.62 years old, mean education ± SD = 13.85 ± 2.94 years, mean HAMD score = 27.86 ± 7.64). The characteristics of the two groups were compared by chi-square test (for gender, family history, and marriage) and independent-sample *t*-test (for age, education, and HAMD score). No significant differences were found between the A and T allele groups. The demographic distribution of the A and T allele groups are shown in Table 3.

**Table 3:** A and T allele demographic distribution and pharmacotherapy.

Variables	A allele (*n* = 58)	T allele (*n* = 100)	*p*-value
Gender (male/female)	17/41	44/56	0.068^a^
Family history (with/without)	45/13	77/23	0.548^a^
Marriage (married/not married)	23/35	42/58	0.453^a^
Age (years)	34.40 ± 11.23	32.60 ± 9.62	0.134^b^
Education (years)	13.09 ± 2.98	13.85 ± 2.94	0.496^b^
HAMD score	27.90 ± 7.01	27.86 ± 7.64	0.372^b^
**Medication treatment (number of patients)**			
SSRI	23	46	–
SNRI	14	21	–
Others	1	2	–
Hypnotics	36	63	–
Medication free	1	2	–
Medication naive	17	35	–

^a^*The *p*-value was obtained by two-tailed chi-square test. ^*b*^Data is presented as mean ± standard deviation. The *p*-value was obtained by two-tailed independent *t*-test*.

**FIGURE 1:**
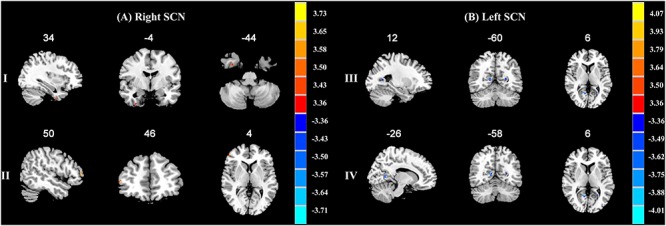
**(A)** Brain regions showing significant functional connectivity differences between A allele and T carrier group with the right suprachiasmatic nuclei (SCN). The clusters with higher functional connectivities are shown in yellow and red (*p* < 0.001, AlphaSim corrected). (I) The right fusiform gyrus. (II) The right middle frontal gyrus. **(B)** Brain regions showing significant functional connectivity differences between A allele and T carrier group with the left suprachiasmatic nuclei (SCN). The clusters with lower functional connectivities are shown in blue (*p* < 0.001, AlphaSim corrected). (III) The right lingual gyrus. (IV) The left calcarine sulcus. The MNI coordinates are shown above each slice.

When compared with the A allele group (*p* < 0.001, *k* = 6 voxels, *p* < 0.05 corrected for multiple comparisons with AlphaSim), increased functional connectivities of the right SCN with the right fusiform gyrus (34, -4, -44; peak *T*-value = 3.5765) and the right middle frontal gyrus (50, 46, 4; peak *T*-value = 3.7615) were observed in the T allele group (see Figure 1A). Additionally, there was a decrease in functional connectivities of the left SCN with the right lingual gyrus (12, -60, 6; peak *T*-value = -4.138) and the left calcarine sulcus in the T allele group (-26, -58, 6; peak T value = -3.7248) (see Figure 1B). The functional connectivities of the left and right SCN are presented in Table 4.

**Table 4:** Functional connectivity of regions of interest (ROIs).

Brain regions	BA	Cluster size	Peak MNI coordinates			Peak *t*-value
			*X*	*Y*	*Z*	
**Right SCN**						
Fusiform_R	20	8	34	-4	-44	3.5765
Frontal_Mid_R	46	6	50	46	4	3.7615
**Left SCN**						
Lingual_R	30	16	12	-60	6	-4.138
Calcarine_L	NA	6	-26	-58	6	-3.7248

*AlphaSim corrected (*p <* 0.001). BA, Brodmann’s area*.

## Discussion

In this study, TPH-2 gene variants and its associated functional brain changes were investigated in early wakening MDD patients. We found that rs4290270 (A/T allele) was linked to the early waking symptom in MDD patients. The differences in functional connectivities in the right fusiform gyrus, right middle frontal gyrus, right lingual gyrus, and left calcarine sulcus with regard to the both the right and left SCN when comparing the A and T allele groups, corresponded to brain regions which are related to visual processing and sleep problems. These results provided a novel insight on sleep-related circadian rhythm disturbances in MDD patients.

rs4290270 was prominently associated to the early wakening MDD group rather than the whole MDD group and non-early wakening MDD group. This result was consistent with a previous study which reported that rs4290270 served as a biomarker for depression patients with primary insomnia in Han Chinese population (Mei et al., 2018). In the past, rs4290270 showed an association between symptoms of disturbed sleep and MDD (Utge, 2012). However, Shen et al. (2011) reported a negative association between rs4290270 of TPH-2 and MDD. Our result was partly consistent with Shen et al. because the whole MDD group had no association with rs4290270 whereas the early wakening MDD group was prominently linked with rs4290270. rs4290270 is located in exon 9 of TPH2 and the ancestral allele was denoted as A in the dbSNP database. In our results, the frequency of T allele was higher in MDD patients compared to HC, especially in the WE group (*T* = 0.6057, *A* = 0.3943). A previous study, which examined the allelic expression of TPH-2 mRNA in human brain, disclosed that rs4290270 acted as a functional *cis*-acting polymorphism due to increased TPH-2 expression levels (Lim et al., 2006). Furthermore, the expression of the minor allele (T allele) exceeded that of the major allele (A allele), potentially suggesting that this variant might be functional.

rs4290270 was also associated with depressive mood and suicide in previous studies. The TT homozygotes were reported to affect the splicing and editing of TPH-2 pre-mRNAs in suicide attempters who experienced childhood general traumas, thereby increasing the risk for suicide in psychiatric patients (Karanovic et al., 2017). Furthermore, a study on the subjective effects of cocaine suggested that the rs4290270 TT homozygote might be associated with depressive mood or suicide (Patriquin et al., 2017). Moreover, rs4290270 had a major effect on the cognitive function of Chinese Han individuals who suffered from late-onset depression (Wang et al., 2015). At present, there is a paucity of studies regarding the relationship between rs4290270 and circadian rhythm.

After confirming the presence of rs4290270 in early wakening MDD patients, the resting-state functional connectivity of the SCN was estimated between A and T allele groups. The right SCN had an increased functional connectivity in the right fusiform gyrus and right middle frontal gyrus whereas the left SCN had a decreased functional connectivity in the right lingual gyrus and left calcarine sulcus in the T allele group. Our results demonstrated functional connectivity abnormalities in the brain regions including the fusiform gyrus, lingual gyrus, and calcarine sulcus, which are all closely linked to visual processing (Prasad and Galetta, 2011). In addition, these regions also have notable findings concerning sleep disturbances.

Environmental light, as a zeitgeber, is involved in visual processing and plays an important role in the synchronization of circadian rhythms in the SCN. Given that blind individuals, with no light exposure or perception, experienced circadian disruption, and severe sleep disturbances, (Skene et al., 1999) the brain areas linked to visual processing are implicated in the maintenance of sleep-related circadian rhythms.

The fusiform gyrus is normally involved in high-level visual processing including recognition of faces, color and words. In another study, healthy subjects with sleep deprivation and post-sleep recovery reported a significant decline in brain activity in the right fusiform gyrus (Bell-McGinty et al., 2004). There was a decrease in glucose metabolism in the fusiform gyrus of MDD patients with sleep disturbances (Nofzinger et al., 2005). The central histaminergic neuron system, which is responsible for regulating wakefulness and sleep-awake cycle, had reduced binding potential values of [11C]-doxepin in bilateral middle frontal gyrus and right lingual gyrus of depressed patients (Kano et al., 2004). In MDD patients, there was a lower cerebral glucose metabolism in the right lingual gyrus after sleep deprivation (Wu et al., 2008). The aforementioned findings indicated that the brain regions involved with early wakening overlapped with those associated to visual processing, suggesting that the desynchronization of these brain regions could be a cue for the sleep-related circadian disruption.

Although the right middle frontal gyrus is mainly associated with attention, emotional processing, and hyperarousal states (Zhou et al., 2017), it had prominent implications in sleep disturbances. There was a decline in glucose metabolism in the middle frontal gyrus after sleep deprivation. After sleep recovery, glucose metabolism was increased in the middle frontal gyrus (Wu et al., 2006). Since there were numerous findings in relation to sleep problems in the middle frontal gyrus and our results showed an altered functional connectivity between the right SCN and the right middle frontal gyrus in early awakening MDD patients, it could be inferred that the right middle frontal gyrus is potentially involved in sleep-related circadian disruption, especially early wakening.

The main limitation of the current study is that sample size is small. In order to minimize the influence of small sample size, we chose the SNPs which were reported in the previous studies, and performed an independent sample verification in our sample. In addition, neuroimaging techniques like rs-fMRI require drastically fewer subjects (tens versus hundreds) compared to other methods for the identification of candidate gene effects on the brain (Hariri and Weinberger, 2003). Another limitation is the effect of medication on brain function. We reduced the impact of medication by arranging the scan 1 or 2 days after hospitalization (see details about medications in Table 3). Although medications have no influence over genetic variants, there is a slight possibility that they could affect our results.

## Conclusion

The TPH-2 gene variant, rs4290270, was observed in early wakening MDD patients. The T allele of rs4290270 demonstrated a higher functional connectivity in the right fusiform gyrus and right middle frontal gyrus from the right SCN and a lower functional connectivity in the right lingual gyrus and left calcarine sulcus from the left SCN. Our findings revealed that the brain regions involved with early wakening overlapped with those associated to visual processing. Since sleep-related circadian disturbances is closely associated to visual processing, there is a possibility that TPH-2 also modulates the visual neural pathway in the SCN of MDD patients, thereby inducing early wakening symptom. Therefore, rs4290270, could serve as a susceptibility variant of sleep-related circadian rhythm disorder in depression. Future investigations could potentially replicate, and hence, validate the involvement of rs4290270 with circadian disruption, in particular early wakening, in MDD.

## Author Contributions

ST designed and performed the experiments. JG and RZ contributed to the clinical data collection and assessment. ST, RY, and JS analyzed the results. ST and MC interpreted the results and wrote the manuscript. ZY and QL overviewed the whole experiments and approved the final manuscript.

## Conflict of Interest Statement

The authors declare that the research was conducted in the absence of any commercial or financial relationships that could be construed as a potential conflict of interest.

## Footnotes

1 https://www.ncbi.nlm.nih.gov/projects/SNP

2 http://broadinstitute.github.io/picard/

3 http://www.mricro.com

4 http://www.restfmri.net/forum/DPARSF

5 http://www.restfmri.net/forum/REST

## References

[B1] Anttila S. Viikki M. Huuhka K. Huuhka M. Huhtala H. Rontu R. (2009). TPH2 polymorphisms may modify clinical picture in treatment-resistant depression. *Neurosci. Lett.* 464 43–46. 10.1016/j.neulet.2009.08.018 19679166

[B2] Association A. P. Association A. P. (2000). *DSM-IV-TR: Diagnostic and Statistical Manual of Mental Disorders, Text Revision*, Vol. 75 Washington, DC: American Psychiatric Association, 78–85

[B3] Bach-Mizrachi H. Underwood M. D. Kassir S. A. Bakalian M. J. Sibille E. Tamir H. (2006). Neuronal tryptophan hydroxylase mRNA expression in the human dorsal and median raphe nuclei: major depression and suicide. *Neuropsychopharmacology* 31 814–824. 10.1038/sj.npp.1300897 16192985

[B4] Baron K. G. Reid K. J. (2014). Circadian misalignment and health. *Int. Rev. Psychiatry* 26 139–154. 10.3109/09540261.2014.911149 24892891 PMC4677771

[B5] Bell-McGinty S. Habeck C. Hilton H. J. Rakitin B. Scarmeas N. Zarahn E. (2004). Identification and differential vulnerability of a neural network in sleep deprivation. *Cereb. Cortex* 14 496–502. 10.1093/cercor/bhh011 15054065

[B6] Bunney B. G. Li J. Z. Walsh D. M. Stein R. Vawter M. P. Cartagena P. (2015). Circadian dysregulation of clock genes: clues to rapid treatments in major depressive disorder. *Mol. Psychiatry* 20 48–55. 10.1038/mp.2014.138 25349171 PMC4765913

[B7] Buysse D. J. (2013). Insomnia. *JAMA* 309 706–716. 10.1001/jama.2013.193 23423416 PMC3632369

[B8] Ciarleglio C. Resuehr H. McMahon D. (2011). Interactions of the serotonin and circadian systems: nature and nurture in rhythms and blues. *Neuroscience* 197 8–16. 10.1016/j.neuroscience.2011.09.036 21963350

[B9] Dagan Y. (2002). Circadian rhythm sleep disorders (CRSD). *Sleep Med. Rev.* 6 45–55. 10.1053/smrv.2001.0190 12531141

[B10] Edgar N. McClung C. A. (2013). Major depressive disorder: a loss of circadian synchrony? *Bioessays* 35 940–944. 10.1002/bies.201300086 24003004 PMC4066996

[B11] Gao J. Pan Z. Jiao Z. Li F. Zhao G. Wei Q. (2012). TPH2 gene polymorphisms and major depression–a meta-analysis. *PLoS One* 7:e36721. 10.1371/journal.pone.0036721 22693556 PMC3365065

[B12] Haghighi F. Bach-Mizrachi H. Huang Y. Arango V. Shi S. Dwork A. (2008). Genetic architecture of the human tryptophan hydroxylase 2 gene: existence of neural isoforms and relevance for major depression. *Mol. Psychiatry* 13 813–820. 10.1038/sj.mp.4002127 18180764

[B13] Han K. M. Won E. Kang J. Kim A. Yoon H. K. Chang H. S. (2017). Local gyrification index in patients with major depressive disorder and its association with tryptophan hydroxylase-2 (TPH2) polymorphism. *Hum. Brain Mapp.* 38 1299–1310. 10.1002/hbm.23455 27807918 PMC6866875

[B14] Hariri A. R. Weinberger D. R. (2003). Imaging genomics. *Br. Med. Bull.* 65 259–270. 10.1093/bmb/65.1.259 12697630

[B15] Hergueta T. Baker R. Dunbar G. C. (1998). The Mini-International Neuropsychiatric Interview (MINI): the development and validation of a structured diagnostic psychiatric interview for DSM-IVand ICD-10. *J. Clin. Psychiatry* 59(Suppl. 20), 22–33. 9881538

[B16] Hickie I. B. Naismith S. L. Robillard R. Scott E. M. Hermens D. F. (2013). Manipulating the sleep-wake cycle and circadian rhythms to improve clinical management of major depression. *BMC Med.* 11:79. 10.1186/1741-7015-11-79 23521808 PMC3760618

[B17] Hickie I. B. Rogers N. L. (2011). Novel melatonin-based therapies: potential advances in the treatment of major depression. *Lancet* 378 621–631. 10.1016/S0140-6736(11)60095-0 21596429

[B18] Jacobsen J. P. R. Siesser W. B. Sachs B. D. Peterson S. Cools M. J. Setola V. (2011). Deficient serotonin neurotransmission and depression-like serotonin biomarker alterations in tryptophan hydroxylase 2 (Tph2) loss-of-function mice. *Mol. Psychiatry* 17 694. 10.1038/mp.2011.50 21537332 PMC3536482

[B19] Kano M. Fukudo S. Tashiro A. Utsumi A. Tamura D. Itoh M. (2004). Decreased histamine H1 receptor binding in the brain of depressed patients. *Eur. J. Neurosci.* 20 803–810. 10.1111/j.1460-9568.2004.03540.x 15255990

[B20] Karanovic J. Ivkovic M. Jovanovic V. M. Svikovic S. Pantovic-Stefanovic M. Brkusanin M. (2017). Effect of childhood general traumas on suicide attempt depends on TPH2 and ADARB1 variants in psychiatric patients. *J. Neural Transm.* 124 621–629. 10.1007/s00702-017-1677-z 28084537

[B21] Ke L. Qi Z. Y. Ping Y. Ren C. Y. (2006). Effect of SNP at position 40237 in exon 7 of the TPH2 gene on susceptibility to suicide. *Brain Res.* 1122 24–26. 10.1016/j.brainres.2006.09.007 17011525

[B22] Lehto K. Vaht M. Mäestu J. Veidebaum T. Harro J. (2015). Effect of tryptophan hydroxylase-2 gene polymorphism G-703 T on personality in a population representative sample. *Prog. Neuro Psychopharmacol. Biol. Psychiatry* 57 31–35. 10.1016/j.pnpbp.2014.10.005 25455586

[B23] LimJ. E.PinsonneaultJ.SadeeW.SaffenD. (2006). Tryptophan hydroxylase 2 (TPH2) haplotypes predict levels of TPH2 mRNA expression in human pons. *Mol. Psychiatry* 12 491–501. 10.1038/sj.mp.4001923 17453063

[B24] Lovenberg T. W. Baron B. M. de Lecea L. Miller J. D. Prosser R. A. Rea M. A. (1993). A novel adenylyl cyclase-activating serotonin receptor (5-HT7) implicated in the regulation of mammalian circadian rhythms. *Neuron* 11 449–458. 10.1016/0896-6273(93)90149-L 8398139

[B25] Major Depressive Disorder Working Group of the Psychiatric Genomics Consortium (2012). A mega-analysis of genome-wide association studies for major depressive disorder. *Mol. Psychiatry* 18 497–511. 10.1038/mp.2012.21 22472876 PMC3837431

[B26] Malek Z. S. Pevet P. Raison S. (2004). Circadian change in tryptophan hydroxylase protein levels within the rat intergeniculate leaflets and raphe nuclei. *Neuroscience* 125 749–758. 10.1016/j.neuroscience.2004.01.031 15099688

[B27] Mandelli L. Antypa N. Nearchou F. A. Vaiopoulos C. Stefanis C. N. Serretti A. (2012). The role of serotonergic genes and environmental stress on the development of depressive symptoms and neuroticism. *J. Affect. Disord.* 142 82–89. 10.1016/j.jad.2012.03.047 22868061

[B28] Martinowich K. Lu B. (2007). Interaction between BDNF and serotonin: role in mood disorders. *Neuropsychopharmacology* 33 73–83. 10.1038/sj.npp.1301571 17882234

[B29] Mei F. Wu Y. Wu J. (2018). Association of tryptophan hydroxylase-2 gene rs4290270 with primary insomnia and depressive symptoms in a chinese han population. *Balkan Med. J.* 10.4274/balkanmedj.2017.1406 [Epub ahead of print]. 29952309 PMC6251380

[B30] Morin L. P. (2013). Neuroanatomy of the extended circadian rhythm system. *Exp. Neurol.* 243 4–20. 10.1016/j.expneurol.2012.06.026 22766204 PMC3498572

[B31] Nofzinger E. A. Buysse D. J. Germain A. Price J. C. Meltzer C. C. Miewald J. M. (2005). Alterations in regional cerebral glucose metabolism across waking and non–rapid eye movement sleep in depression. *Arch. Gen. Psychiatry* 62 387–396. 10.1001/archpsyc.62.4.387 15809406

[B32] Ottenhof K. W. Sild M. Lévesque M. L. Ruhé H. G. Booij L. (2018). TPH2 polymorphisms across the spectrum of psychiatric morbidity: a systematic review and meta-analysis. *Neurosci. Biobehav. Rev.* 92 29–42. 10.1016/j.neubiorev.2018.05.018 29775696

[B33] Patriquin M. A. Hamon S. C. Harding M. J. Nielsen E. M. Newton T. F. De La Garza R. (2017). Genetic moderation of cocaine subjective effects by variation in the TPH1, TPH2, and SLC6A4 serotonin genes. *Psychiatr. Genet.* 27 178–186. 10.1097/YPG.0000000000000178 28590957 PMC5572746

[B34] Piel J. Lett T. Wackerhagen C. Plichta M. Mohnke S. Grimm O. (2018). The effect of 5-HTTLPR and a serotonergic multi-marker score on amygdala, prefrontal and anterior cingulate cortex reactivity and habituation in a large, healthy fMRI cohort. *Eur. Neuropsychopharmacol.* 28 415–427. 10.1016/j.euroneuro.2017.12.014 29358097

[B35] Prasad S. Galetta S. L. (2011). Anatomy and physiology of the afferent visual system. *Handb. Clin. Neurol.* 102 3–19. 10.1016/B978-0-444-52903-9.00007-8 21601061

[B36] Reichert C. F. Maire M. Gabel V. Viola A. U. Götz T. Scheffler K. (2017). Cognitive brain responses during circadian wake-promotion: evidence for sleep-pressure-dependent hypothalamic activations. *Sci. Rep.* 7:5620. 10.1038/s41598-017-05695-1 28717201 PMC5514145

[B37] Saletu-Zyhlarz G. M. Abu-Bakr M. H. Anderer P. Gruber G. Mandl M. Strobl R. (2002). Insomnia in depression: differences in objective and subjective sleep and awakening quality to normal controls and acute effects of trazodone. *Prog. Neuro Psychopharmacol. Biol. Psychiatry* 26 249–260. 10.1016/S0278-5846(01)00262-7 11817501

[B38] Serretti A. Chiesa A. Porcelli S. Han C. Patkar A. A. Lee S.-J. (2011). Influence of TPH2 variants on diagnosis and response to treatment in patients with major depression, bipolar disorder and schizophrenia. *Psychiatry Res.* 189 26–32. 10.1016/j.psychres.2011.02.001 21396719

[B39] Shen X. Wu Y. Qian M. Wang X. Hou Z. Liu Y. (2011). Tryptophan hydroxylase 2 gene is associated with major depressive disorder in a female Chinese population. *J. Affect. Disord.* 133 619–624. 10.1016/j.jad.2011.04.037 21620479

[B40] Skene D. J. Lockley S. W. Thapan K. Arendt J. (1999). Effects of light on human circadian rhythms. *Reprod. Nutr. Dev.* 39 295–304. 10.1051/rnd:19990302 10420432

[B41] Soehner A. M. Kaplan K. A. Harvey A. G. (2014). Prevalence and clinical correlates of co-occurring insomnia and hypersomnia symptoms in depression. *J. Affect. Disord.* 167 93–97. 10.1016/j.jad.2014.05.060 24953480 PMC4291280

[B42] Tsai S.-J. Hong C.-J. Liou Y.-J. Younger W. Chen T.-J. Hou S.-J. (2009). Tryptophan hydroxylase 2 gene is associated with major depression and antidepressant treatment response. *Prog. Neuro Psychopharmacol. Biol. Psychiatry* 33 637–641. 10.1016/j.pnpbp.2009.02.020 19272410

[B43] Utge S. J. (2012). *A Study of Candidate Genes in Depression and Disturbed Sleep.* Ph. D. Dissertation, National Institute for Health and Welfare (THL), Helsinki, 63–69.

[B44] Vadnie C. A. McClung C. A. (2017). Circadian rhythm disturbances in mood disorders: insights into the role of the suprachiasmatic nucleus. *Neural Plast.* 2017:1504507. 10.1155/2017/1504507 29230328 PMC5694588

[B45] Van der Auwera S. Janowitz D. Schulz A. Homuth G. Nauck M. Völzke H. (2014). Interaction among childhood trauma and functional polymorphisms in the serotonin pathway moderate the risk of depressive disorders. *Eur. Arch. Psychiatry Clin. Neurosci.* 264 45–54. 10.1007/s00406-014-0536-2 25214390

[B46] Vimal R. L. Pandey-Vimal M. U. Vimal L. S. Frederick B. B. Stopa E. G. Renshaw P. F. (2009). Activation of suprachiasmatic nuclei and primary visual cortex depends upon time of day. *Eur. J. Neurosci.* 29 399–410. 10.1111/j.1460-9568.2008.06582.x 19200242

[B47] Vitaterna M. H. Takahashi J. S. Turek F. W. (2001). Overview of circadian rhythms. *Alcohol Res. Health* 25 85–93. 11584554 PMC6707128

[B48] Wang X. Wang Z. Wu Y. Hou Z. Yuan Y. Hou G. (2015). Tryptophan hydroxylase 2 gene is associated with cognition in late-onset depression in a Chinese Han population. *Neurosci. Lett.* 600 98–103. 10.1016/j.neulet.2015.06.010 26057341

[B49] Whitney M. S. Shemery A. M. Yaw A. M. Donovan L. J. Glass J. D. Deneris E. S. (2016). Adult brain serotonin deficiency causes hyperactivity, circadian disruption, and elimination of siestas. *J. Neurosci.* 36 9828–9842. 10.1523/JNEUROSCI.1469-16.2016 27656022 PMC5030349

[B50] Wigner P. Czarny P. Synowiec E. Bijak M. Białek K. Talarowska M. (2018). Association between single nucleotide polymorphisms of TPH1 and TPH2 genes, and depressive disorders. *J. Cell Mol. Med.* 22 1778–1791. 10.1111/jcmm.13459 29314569 PMC5824396

[B51] Wirz-Justice A. (2003). Chronobiology and mood disorders. *Dialogues Clin. Neurosci.* 5 315–325. 22033593 10.31887/DCNS.2003.5.4/awirzjustice PMC3181777

[B52] World Health Organization [WHO] (2017). *WHO Depression Fact Sheet.* Geneva: WHO.

[B53] World Medical Association (2013). World medical association declaration of helsinki: ethical principles for medical research involving human subjects. *JAMA* 310 2191–2194. 10.1001/jama.2013.281053 24141714

[B54] Wu J. C. Gillin J. C. Buchsbaum M. S. Chen P. Keator D. B. Wu N. K. (2006). Frontal lobe metabolic decreases with sleep deprivation not totally reversed by recovery sleep. *Neuropsychopharmacology* 31:2783. 10.1038/sj.npp.1301166 16880772

[B55] Wu J. C. Gillin J. C. Buchsbaum M. S. Schachat C. Darnall L. A. Keator D. B. (2008). Sleep deprivation PET correlations of Hamilton symptom improvement ratings with changes in relative glucose metabolism in patients with depression. *J. Affect. Disord.* 107 181–186. 10.1016/j.jad.2007.07.030 18031825

[B56] Xu X. Ding M. Pang H. Xing J. Xuan J. Wang B. (2013). Genetic polymorphisms of SNP loci in the 5′ and 3′ region of TPH2 gene in Northern Chinese Han population. *Fa Yi Xue Za Zhi* 29 21–24. 23646497

[B57] Xu Z. Reynolds G. P. Yuan Y. Shi Y. Pu M. Zhang Z. (2016). TPH-2 polymorphisms interact with early life stress to influence response to treatment with antidepressant drugs. *Int. J. Neuropsychopharmacol.* 19 yw070. 10.1093/ijnp/pyw070 27521242 PMC5137282

[B58] Zhang X. Beaulieu J. M. Sotnikova T. D. Gainetdinov R. R. Caron M. G. (2004). Tryptophan hydroxylase-2 controls brain serotonin synthesis. *Science* 305:217. 10.1126/science.1097540 15247473

[B59] Zhou F. Huang S. Zhuang Y. Gao L. Gong H. (2017). Frequency-dependent changes in local intrinsic oscillations in chronic primary insomnia: a study of the amplitude of low-frequency fluctuations in the resting state. *Neuroimage Clin.* 15 458–465. 10.1016/j.nicl.2016.05.011 28649490 PMC5470569

[B60] Zill P. Baghai T. Zwanzger P. Schüle C. Eser D. Rupprecht R. (2004). SNP and haplotype analysis of a novel tryptophan hydroxylase isoform (TPH2) gene provide evidence for association with major depression. *Mol. Psychiatry* 9 1030–1036. 10.1038/sj.mp.4001525 15124006

